# Trihydrogen Formation on Gold Nanoparticles in Strong Laser Fields

**DOI:** 10.1021/acs.nanolett.5c03438

**Published:** 2026-01-27

**Authors:** Ritika Dagar, Wenbin Zhang, Philipp Rosenberger, Marcel Neuhaus, Boris Bergues, Cesar Costa Vera, Matthias F. Kling

**Affiliations:** † Department of Physics, 130455 Ludwig-Maximilians-Universität Munich, D-85748 Garching, Germany; ‡ Max Planck Institute of Quantum Optics, D-85748 Garching, Germany; ¶ Stanford PULSE Institute, SLAC National Accelerator Laboratory, Menlo Park, California 94025, United States; § State Key Laboratory of Precision Spectroscopy, 12655 East China Normal University, Shanghai 200241, China; ∥ Department of Physics, 27882 Escuela Politecnica Nacional, Quito 170525, Ecuador; ⊥ Applied Physics Department, 6429 Stanford University, Stanford, California 94305, United States

**Keywords:** gold nanoparticles, facets, reactivity, surface emission, reaction nanoscopy

## Abstract

The trihydrogen cation (H_3_^+^) plays a central role in proton-transfer chemistry, astrochemical pathways, and hydrogen plasma environments, acting as a key indicator of ultrafast proton rearrangement. Although H_3_^+^ formation has been studied extensively in the gas phase, its surface-mediated generation and its sensitivity to nanoparticle morphology remain largely unexplored. Gold nanoparticles (AuNPs), which can localize surface charge and sustain strong electric fields, offer an ideal platform to probe such nonequilibrium reaction pathways. Using reaction nanoscopy, we spatially map H_3_^+^ production on AuNPs exposed to intense femtosecond laser fields. By comparing spherical and faceted nanoparticles, we demonstrate how morphology modulates the charge density and governs the reaction efficiency. We find that sharp features on faceted particles concentrate charge more effectively, promoting molecular fragmentation and enabling proton rearrangement and migration that enhance H_3_^+^ yields. This work opens new directions for exploiting strong-field interactions at metal interfaces to drive nanoscale reactivity and photocatalysis.

## Graphical abstract

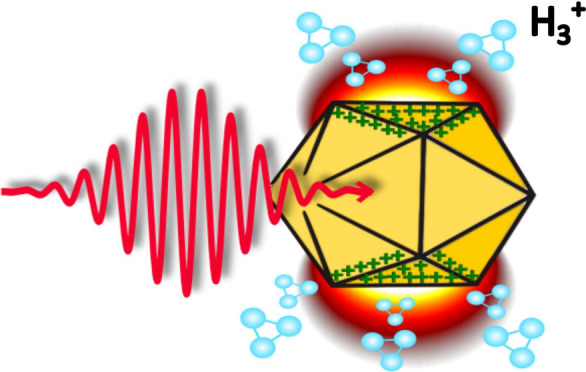

Strong electric fields arising from localized surface charge densities on nanoparticles can dramatically reshape catalytic environments, driving ultrafast surface reactions and fragmentation dynamics.^1^ Recent time-resolved measurements have shown that surface charge dynamics can directly weaken chemical bonds in surface-adsorbed molecules, highlighting the role of surface charge in dictating nanoscale chemical reactivity.^2^ For instance, surface charging enhances the reductive power of catalysts, promotes CO_2_ activation on supported single-atom catalysts, and underpins synergistic effects in plasma-assisted catalysis.^3^ Consequently, controlling surface charge states has emerged as a promising strategy for tuning catalytic activity and selectivity in diverse applications, ranging from electrocatalysis and photocatalysis to CO oxidation.^4–8^

Gold, though catalytically inert in bulk, displays remarkable reactivity at the nanoscale due to its ability to localize charges and host reactive surface sites. The catalytic behavior of AuNPs is highly sensitive to surface morphology, including the coordination environment of edges, corners, and facets.^9,10^ Prior work has linked enhanced CO oxidation to low-coordination sites and demonstrated size-dependent reactivity trends based on CO adsorption energies.^11^ Further studies^12,13^ showed that both electrostatic interactions and structural motifs, such as spine-like geometries, can influence reaction selectivity and efficiency. Collectively, these findings underscore how nanoparticle morphology governs local charge distributions and, in turn, dictates catalytic behavior. While the catalytic significance of surface morphology and equilibrium charge states is well-known, the ways in which strong field-driven surface charges reshape local chemical environments and influence reactivity on individual nanoparticles remain poorly understood. The formation of H_3_^+^ provides a sensitive probe for ultrafast proton transfer, a key step in a wide range of physical and chemical processes, especially at initial transient stages. As the simplest polyatomic ion, H_3_^+^ plays a central role in interstellar chemistry, hydrogen plasma dynamics, and high-energy reaction environments. Its formation is indicative of highly nonlinear, multicenter rearrangement pathways that lie beyond conventional surface reaction mechanisms. Observing H_3_^+^ on ionized nanoparticle surfaces, therefore provides a unique window into laser-driven surface chemistry and the transient charge states that govern it. The formation of H_3_^+^ offers a powerful route to explore ultrafast, nontraditional chemical pathways relevant to both fundamental and applied science.^14,15^ Previous work has shown that H_3_^+^ can form on silica nanoparticles under intense laser fields.^16^ However, silica-like systems are limited by their low surface charge densities due to their dielectric nature. Here, we employ citrate-functionalized AuNPs, which combine strong field localization with hydrogen-rich surface ligands. This duality enhances proton transfer and facilitates H_3_^+^ generation.

In this work, we generate transient surface charges by employing strong-field laser irradiation and probe their chemical effects via the formation of molecular ions on the surfaces of AuNPs. Previous studies using strong-field ionization of plasmonic nanoparticles primarily measured electron dynamics^17–19^ or mainly focused on imaging the near-field profiles around the nanostructures.^20–22^ However, these approaches fall short of directly capturing surface chemistry or bond-breaking events. Despite their promise, strong-field ionized plasmonic nanoparticles have yet to be fully explored in nanoscale catalysis. In particular, systematic investigations of ion emission, molecular adsorbate fragmentation, and the formation of complex ionic species, such as H_3_^+^, can offer new insights into nanoscale surface chemistry.

To achieve this, we use reaction nanoscopy, an emerging technique that enables spatially resolved, all-optical probing of surface chemical reactions with nanoscale precision.^23,24^ Our approach enables us to directly map reaction yields across individual nanoparticles and resolve how morphology and charge localization control chemical outcomes. Unlike traditional methods, such as super-resolution fluorescence microscopy, which relies on fluorescent labeling^25^ and offers lower temporal resolution, or tip-enhanced Raman spectroscopy (TERS),^26^ which provides high chemical specificity but is limited in speed and surface degree of perturbation, reaction nanoscopy operates label-free and without physical contact, using 3D momentum-resolved ion detection to map reaction products. This makes it ideally suited for exploring how light-matter interactions, surface fields, and chemical environments intersect in photocatalytic nanostructures.^24,27–29^

We spatially map H_3_^+^ formation on both spherical and faceted AuNPs, revealing how surface morphology modulates charge localization and reaction efficiency. We show that high-curvature regions on faceted particles accumulate charge more efficiently, leading to enhanced molecular reactivity. This spatially resolved approach not only identifies active sites at the nanoscale but also provides a general framework for understanding and tuning catalysis in extreme fields. The conceptual framework of this work is illustrated in Figure 1, which highlights the preferential formation of reactive species, such as H_3_^+^, on faceted nanoparticles. The figure shows how surface inhomogeneities on faceted AuNPs cause anisotropic charge localization and strong local field enhancement in contrast to the uniform surface curvature of the spherical particles. The high-curvature features not only modulate the adsorption of surface-bound molecules but also amplify local electric fields under laser excitation, thereby promoting enhanced reactivity.

**Figure 1:**
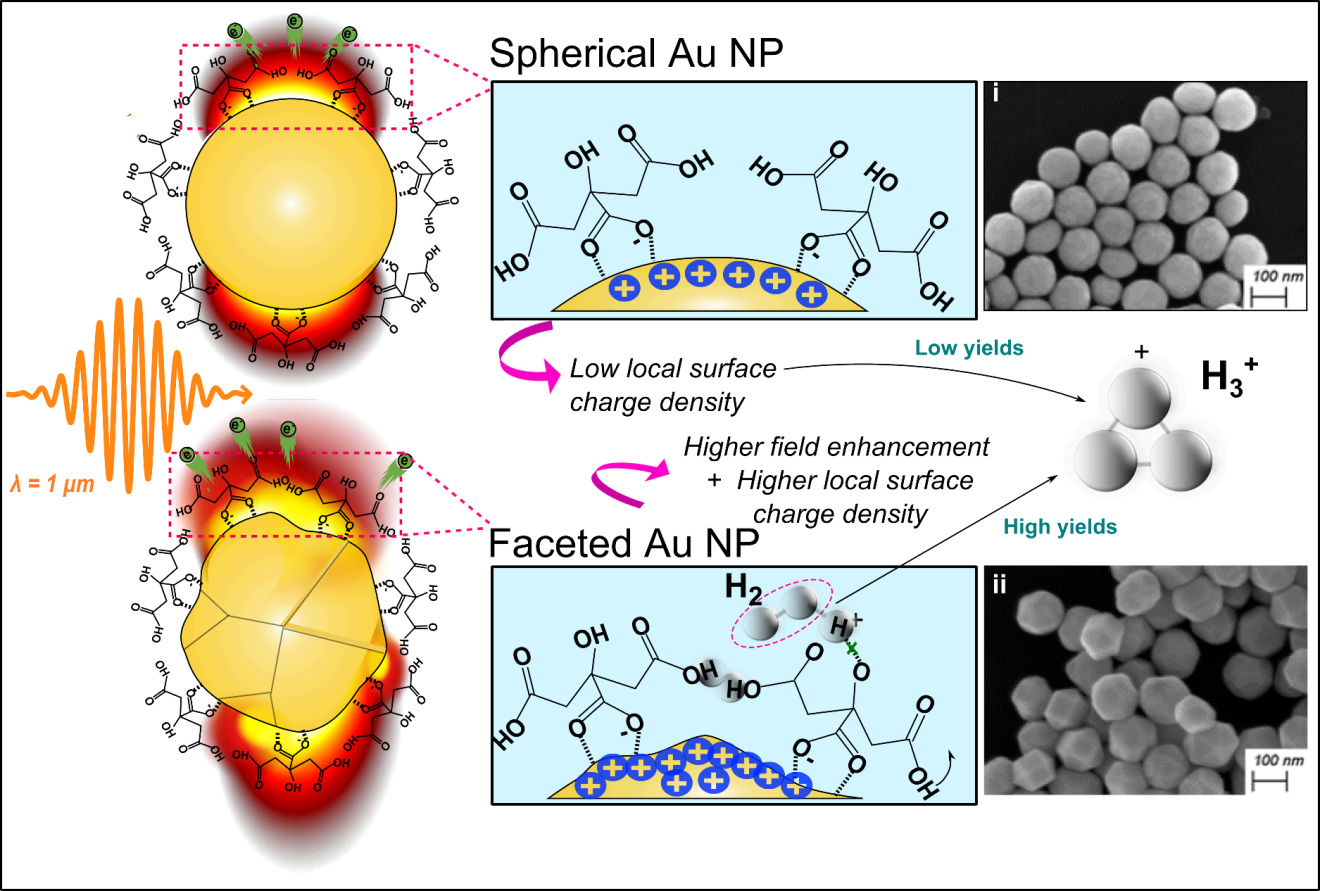
Visualization of the role of surface topography in enhancing the reactivity of AuNPs. This schematic illustrates how the interaction of laser light with spherical and faceted AuNPs, with sizes much smaller than the laser wavelength, is influenced by surface topography. The inhomogeneities present in faceted nanoparticles, such as vertices, edges, and facets, introduce anisotropic surface properties that are absent in spherical nanoparticles. These anisotropies lead to a higher local surface charge density at the high-curvature regions, particularly around the vertices and edges, where the surface experiences stronger electric fields due to plasmonic effects. This increased local charge density not only enhances the local electric field but also promotes preferential interactions between adsorbed molecules, resulting in localized increases in reactivity in faceted AuNPs compared to their spherical counterparts. The SEM images of spherical and faceted AuNPs illustrating the different surface morphologies are shown in insets (i) and (ii), respectively. Further characterization of the AuNPs used in this study is provided in the Supporting Information.

The spherical AuNPs with diameters ∼100 nm were obtained in citrate buffer solution from Nanocomposix, while their faceted counterparts with different sizes (10, 50, 100 nm) were purchased from Sigma-Aldrich. Citrate ions in the buffer solution play a vital role in stabilizing the NPs.^30^ This stabilization mechanism involves the formation of a protective layer around the NPs, preventing their aggregation. In the experiments, the nanoparticle solutions were maintained at the same concentration as purchased, as it was observed that dilution, even with water, resulted in the formation of AuNP aggregates, as verified experimentally. Reaction nanoscopy also permits differentiating between individual nanoparticles and clusters in situ, as previously demonstrated for spherical silica NPs,^27^ thus allowing identifying potentially interfering ionization events.

The experimental setup of reaction nanoscopy has been described in detail in previous work.^23,24,28^ In brief, for this experiment, we used laser pulses with a central wavelength of ∼1 μm and a pulse duration of ∼50 fs from an optical parametric chirped-pulse amplifier laser system with a repetition rate of 100 kHz.^31^ The laser pulses were tightly focused into the reaction nanoscope, achieving intensities of up to 2 × 10^13^ W/cm^2^. The reaction nanoscopy setup involved the aerosolization of AuNPs from an aqueous suspension, followed by their desiccation using a membrane dryer and subsequent collimation via an aerodynamic lens before introduction into the vacuum chamber. Within the interaction region, the intersection of the nanoparticle beam and laser beam resulted in the generation of electrons and ions from the nanoparticle surfaces. These emitted charged species were then detected by two detectors located at opposite ends of the reaction nanoscope time-of-flight spectrometer. Electron hits are recorded using a channeltron detector, which served as a bucket detector to distinguish ionization events from background gas molecules and those resulting from AuNPs. A time- and position-sensitive detector composed of a multichannel plate and delay-line detector, is employed to reconstruct the full three-dimensional (3D) momentum distributions of detected ions.

To simulate the near-field distributions and surface charge densities, we used the finite-difference time-domain (FDTD) solver provided in Lumerical (Ansys Lumerical 2022 R1, version 8.27.2898). To enable a direct comparison of surface reactivity between different nanoparticle geometries, cubic AuNPs were modeled with edge lengths of 7.24, 36.18, and 72.36 nm, corresponding to the surface areas of 10, 50, and 100 nm spherical nanoparticles, respectively. These size labels are used throughout the paper for the sake of clarity and consistency. A regular mesh with a resolution scaled according to particle size and perfectly matched layer (PML) boundary conditions in all directions was implemented. Bulk gold dielectric properties were taken from Johnson and Christy.^32^ The simulated near-field intensities were used to estimate electron ionization probabilities via Fowler-Nordheim (FN) tunneling, appropriate for metallic systems such as AuNPs. Based on the Murphy and Good framework,^33^ with refinements by Forbes,^34^ the tunneling probability was calculated using the JWKB approximation:

1 $$D\approx \exp\left(\frac{-b_{FN}\phi^{3/2}}{E}\right)$$

where

2 $$b_{FN}\approx 6.83089\;{\left[\mathrm{eV}\right]}^{-3/2}\left[V\right]{\left[\mathrm{nm}\right]}^{-1}$$

where *b*_FN_ denotes the Fowler-Nordheim field emission constant, ϕ is the material work function (5.1 eV for bulk gold^32^), and *E* is the local electric field in V/nm. This approach enabled spatial mapping of the ionization likelihood, revealing geometry-induced localization of charges and anisotropic field intensities across the nanoparticle surface.

To convert these dimensionless ionization probabilities into an estimate of surface charge density (in electrons per nm^2^), we implemented the following scheme with normalization:

3 $$\rho_{\mathrm{surface}}=\frac{i_{\mathrm{rate}}\times n_{\mathrm{cand}}}{6L^{2}}\times \frac{n_{\mathrm{Au}}}{\sum \left(i_{\mathrm{rate}}\right)}$$

Here, *i*_rate_ denotes the ionization rate, *L* is the edge length of the nanocube (in nm), and 6*L* ^2^ is the total surface area of all six cube faces. The factor *n*_Au_ = 13.9 atoms/nm^3^ is the atomic number density of bulk gold. This expression scales the dimensionless tunneling probability to yield an estimated number of ionized surface atoms per square nanometer, assuming uniform atomic density and equal contributions from all surface sites.

In line with observations from previous experiments involving water/ethanol-covered SiO_2_ NPs,^23,28^ the ion spectra for citrate-covered AuNPs reveal a dominance of protons obtained from the fragmentation of the citrate molecules. The proton momentum distribution obtained from the laser interaction with spherical AuNPs shows a dipolar distribution along the laser polarization direction, cf. Figure 2a. Such a dipolar emission was observed in numerous previous studies involving spherical SiO_2_ NPs.^23,24,28^ The distribution can be explained with a simple two-step process: (1) the laser creates local surface charges that follow the dipolar field enhancement, and (2) the field (laser + local charges) leads to the generation of protons from the dissociation of surface molecules emitting out radially from the nanoparticle surface.^23,24^ The point-projection feature in reaction nanoscopy enables the final momentum of the emitted ions to be mapped to their birth positions on the nanoparticle surface. Compared with the dipolar emission observed with spherical nanoparticles, as shown in Figure 2b, the momentum distribution of protons from faceted AuNPs exhibits an additional feature. This distribution, obtained from the surface ionization dissociation of citrate molecules adsorbed on the AuNPs, reveals a low-momentum ring when averaged over nanoparticle orientations in the interaction region, alongside the typical high-momentum dipolar structure aligned with the laser’s polarization direction. We attribute the emergence of the low-momentum proton momentum distribution to the inhomogeneous nanoparticle surface. The presence of vertices and edges on nanoparticles leads to stronger local electric fields due to their high curvature, which enhances plasmonic effects. This results in a high local surface charge density generated by the laser, contributing to the observed high proton momentum when the pointed vertices align with the laser polarization direction. In contrast, facets, with their lower curvature, experience weaker field enhancement and charge localization. This results in a lower surface charge density, which leads to protons emitted from these areas experiencing reduced Coulombic repulsion, thus producing a lower momentum distribution. In addition, differences in citrate binding strength across facets and curved surfaces^30^ could contribute to the observed momentum features. Stronger adsorption on flat facets would require more energy for desorption, leading to reduced momentum fragments, consistent with the low-momentum ring observed.

**Figure 2:**
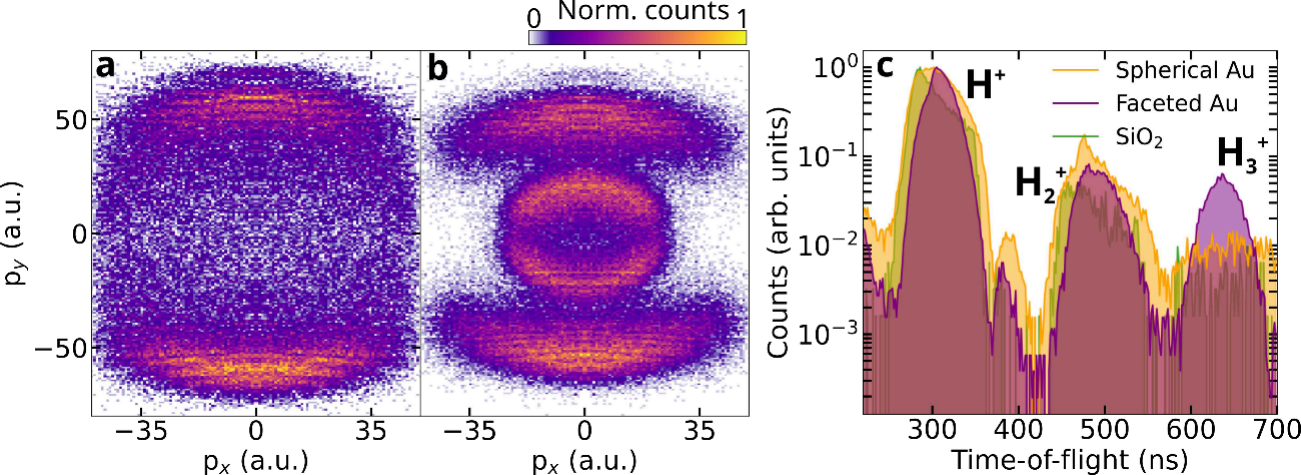
Nanoparticle shape-dependent variation in surface yields. (a,b) Measured proton momentum distribution for 100 nm spherical and faceted AuNPs, respectively. (c) Measured time-of-flight (TOF) spectrum of selected ions emitted from 100 nm spherical and faceted AuNPs in comparison to SiO_2_ NPs.

Simultaneous time-of-flight (TOF) measurements reveal a notable variation in the molecular fragment yields. A significant increase in H_3_^+^ ion yield is observed from citrate-capped AuNPs relative to dielectric silica nanoparticles, as evidenced by the TOF spectrum in Figure 2c. This spectrum also emphasizes the role of the nanoparticle surface heterogeneity in facilitating H_3_^+^ formation from surface molecules in faceted AuNPs. We note that H_3_^+^ formation is observed on both spherical and faceted AuNPs. However, the relative yield is significantly higher for faceted particles, consistent with the presence of localized high-curvature charge sites.

We further analyzed the momentum-resolved distributions of protons from citrate-covered faceted AuNPs of different sizes to investigate the influence of nanoparticle size on strong-field-ionization-induced proton emission. Figures 3a and 3b present the proton momentum distributions in the polarization plane for 50 and 10 nm nanoparticles, respectively. Both figures exhibit a high momentum dipolar structure, with the lower momentum ring attributed to the distinctive presence of surface irregularities. However, as we go lower in AuNP size to 10 nm, the lower momentum ring is found to have vanished, as shown in Figure 3b. This observation correlates with the fact that in larger NPs, various crystallographic features, such as facets, edges, and vertices, become more pronounced. These features are more spatially separated for larger particles, thus allowing a clearer distinction in their contributions to surface effects. Also, as the nanoparticle size decreases, the diminishing surface area occupied by facets makes it harder to isolate their effects from those of the edges or vertices. As a result, the roles of these individual features become intertwined, and their distinct contributions to surface characteristics become less distinguishable as the nanoparticle size decreases. This is consistent with the increased field gradients expected near sharper geometric features and reduced radii of curvature. Additionally, as nanoparticle size decreases, the momenta of emitted protons also decrease, as shown in the comparison between Figures 4a and 4b. This effect is directly linked to the reduced Coulombic force acting on the departing protons, resulting from a lower total global surface charge. It is important to distinguish between global surface charge and local surface charge density; although smaller nanoparticles possess less total charge, their high-curvature regions still display orders-of-magnitude greater local charge density compared with larger particles. These stronger localized fields promote molecular rearrangement, thereby explaining the enhanced H_3_^+^ yield observed in smaller faceted particles (Figure 4d).

Comparative analysis of ion yield distributions for the different sizes of faceted AuNPs reveals contrasting yields of H_3_^+^ molecular ions. As the size of the NPs decreases, the surface-to-volume ratio increases, exposing a larger number of catalytically active sites that can profoundly influence the nanoparticle surface chemistry. This variation in exposed active sites can result in discernible differences in the reactivity of the NPs depending on their sizes. Figure 3c shows the TOF spectra for hydrogen ions with respect to their position (along the laser-polarization direction, *y*) for faceted AuNPs of sizes 100, 50, and 10 nm. The observed asymmetry in the recorded fragments, mostly visible for the proton distribution along the TOF axis, is attributed to a decrease in the detector efficiency for ions arriving later within a mass peak. This reduction in efficiency occurs when ions hit the detector within the recovery time of the microchannel plate. The increase in yields for H_2_^+^ and H_3_^+^ fragments with decreasing AuNP sizes is visible, also highlighted by the pink dashed box enclosing the H_3_^+^ ions. The quantitative analysis of this reduction in fragment yields, specifically concerning the formation of H_3_^+^ as a function of the nanoparticle size, is presented in Figure 3d. Protons emerge as the most abundant fragment species for all three sizes, followed by H_2_^+^ and then H_3_^+^, as is evident in the figure. While the H^+^ yield remains relatively constant across sizes, the molecular ions, particularly H_3_^+^, exhibit a clear enhancement for smaller nanoparticles. The observed yield trend underscores the size- and morphology-dependent nature of bond rearrangement processes under intense local charge densities. The nonlinear dependence of H_3_^+^ formation suggests a cooperative effect involving localized ionization hotspots, which become more prominent as nanoparticle features become sharper and more confined.

**Figure 3:**
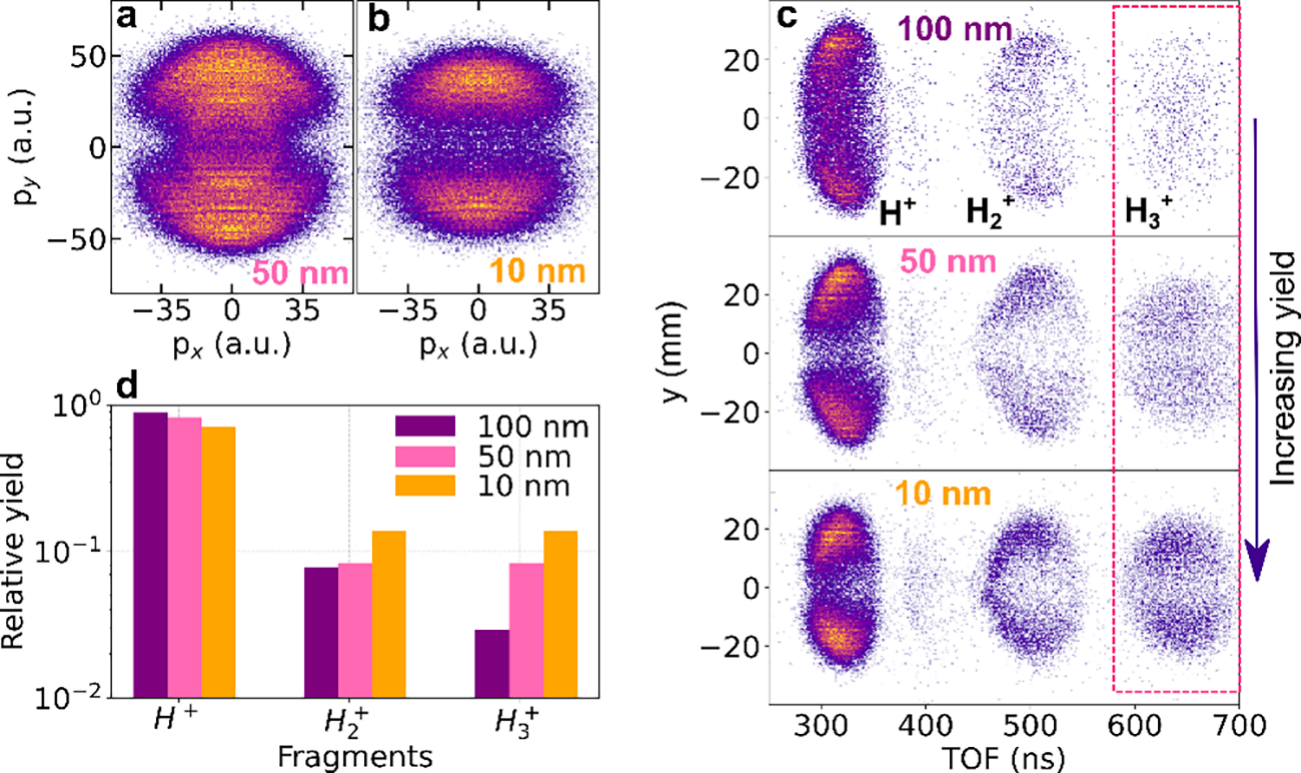
Nanoparticle size-dependent variation in surface yields. (a,b) Measured proton momentum distribution for 50 and 10 nm AuNPs, respectively. (c) Measured time-of-flight (TOF) spectra of the hydrogen ions as a function of the *x*-position of ion fragments emitted from surface molecules on differently sized NPs. The pink dashed box highlights the occurrence of H_3_^+^ and the corresponding increase in the yield as the nanoparticle size decreases. (d) The hydrogen ion yields for different AuNP sizes. The yield is normalized to the total number of counts of all of the fragments for the respective nanoparticle size, shown on a logarithmic scale.

To elucidate how nanoparticle geometry influences ionization behavior under intense laser fields, we conducted numerical simulations of surface charge distributions on model gold nanocubes. These simulations isolate geometric effects on charge localization using idealized cubes as representative models for more intricate faceted nanoparticles. Although the faceted particles in Figure 1 have complex geometries, the cube model provides clear insight into how sharp features govern local field enhancements and charge dynamics. Truncated octahedral or cuboctahedral models would better capture the experimental mixture of {100} and {111} facets, but the cube approximation isolates the role of geometric singularities (corners and edges) that dominate charge localization. Figures 4a−4c present the resulting surface charge density distributions for cubic AuNPs for sizes 10, 50, and 100 nm, respectively. Figures 4a–4c display 3D colormaps of the surface charge density ρ (in e/nm^2^) projected onto the isosurfaces of gold nanocubes with increasing sizes. The color scale reflects the magnitude of the charge density localized at different points on the surface. For the smallest nanocube (10 nm), Figure 4a reveals sharp charge localization at the corners and edges, with maximum ρ values exceeding 0.4 e/nm^2^. As the size increases, the spatial localization becomes increasingly delocalized: the 50 nm nanocube (Figure 4b) shows peak values around 0.01 e/nm^2^, and the 100 nm nanocube (Figure 4c) further drops below 0.005 e/nm^2^. Figure 4d quantifies the distribution of these charge densities across the entire surface by plotting the voxel count versus ρ on a logarithmic scale. For the 10 nm AuNC, a wide range of ρ values is observed, with a high population of surface voxels exhibiting low-to-intermediate charge densities, while still retaining a significant number of high-ρ voxels (>0.3 e/nm^2^). On the other hand, the larger cubes are highly skewed toward low-density voxels, confirming the visual trend in Figures 4a–4c. To assess how these charges are angularly distributed, Figure 4e presents the weighted angular charge density as a function of polar angle θ, with binning performed over surface voxels. The term “weighted” here refers to the summation of local ρ values in each angular bin, effectively representing the directional preference for charge buildup. All nanocube sizes show a U-shaped angular profile with enhanced densities near the cube corners (θ ≈ π/4 and θ ≈ 3π/4), reflecting the geometric field concentration. However, the magnitude and sharpness of this anisotropy strongly depend on size. The 10 nm AuNC displays the most pronounced angular peaks, while the 100 nm cube’s distribution is relatively flat and subdued.

The simulations reveal a consistent trend across all sizes: charge accumulation is highly localized at the corners and edges of the cubes. This behavior is attributed to the presence of geometric singularities, points of abrupt curvature where the electric field tends to concentrate due to boundary conditions imposed on the metallic surface. The degree of charge localization was found to vary markedly with the particle size. In the smallest cube (10 nm), the effect is particularly pronounced. As shown in Figure 4, the surface charge density is significantly elevated at the cube’s corners, indicating strong local field enhancement. In contrast, for the 50 and 100 nm cubes (Figures 4b and 4c), while corner and edge enhancement is still evident, the intensity of the accumulation diminishes. This can be understood in terms of the local radius of curvature. For smaller cubic nanoparticles, the corners exhibit a very small radius of curvature, leading to a high concentration of electric field at these sites. As the particle size increases, the sharpness of these corners diminishes (larger radius of curvature), leading to a more distributed charge accumulation across the surface.

**Figure 4:**
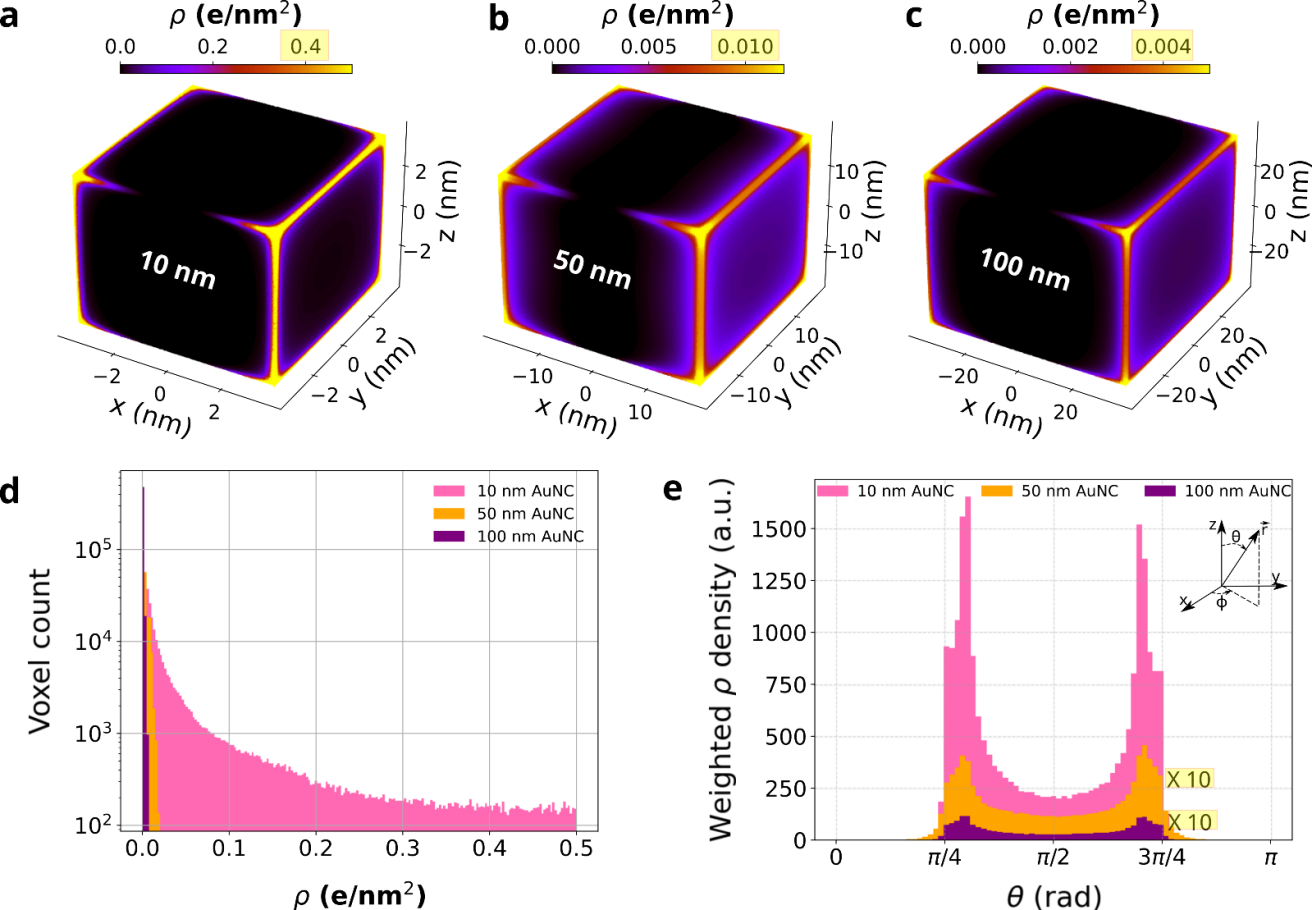
Surface charge density distributions and anisotropy analysis for gold nanocubes of varying sizes. (a–c) Simulated spatial distribution of surface charge density on AuNPs of sizes (a) 10 nm, (b) 50 nm, and (c) 100 nm, respectively, under identical excitation conditions. Charge accumulation is prominently localized at the corners and edges, with intensity decreasing as the nanoparticle size increases. Note that the colorbars are independently rescaled in each panel for visibility; the maximum ρ values are ∼0.48 e/nm^2^ for (a), ∼0.011 e/nm^2^ for (b), and ∼0.0044 e/nm^2^ for (c), highlighting the strong size dependence of charge localization. (d) Histogram of voxel-wise ρ values for 10 nm (pink), 50 nm (orange), and 100 nm (purple) AuNCs, revealing a size-dependent reduction in high-density features. (e) Polar distribution of weighted ρ as a function of polar angle θ from the nanoparticle surface normal, illustrating anisotropic charge localization. Data for 50 and 100 nm AuNCs are scaled by a factor of 10 for visibility. Enhanced localization near θ ≈ π/4 and θ ≈ 3π/4 reflects strong field enhancement at high-curvature sites such as edges and corners, particularly in smaller AuNCs.

In addition to geometric effects, the citrate adsorption varies across surface sites. In faceted AuNPs, lower-coordination sites (steps, edges, corners) and {100} terraces bind more strongly than {111} terraces, while ‘spherical’ particles exhibit stepped microfacets. In the strong-field regime, such variations shift desorption thresholds and redistribute ion momenta, eg., contributing to the low-momentum ring, but they do not change the main trend: localized charge at high-curvature features enhances H_3_^+^ formation. While mixed-facet models would refine quantitative yields, the qualitative conclusion remains robust. Localized fields at sharp features nonlinearly promote H_3_^+^ production, consistent with recent near-field mapping studies.^20–22^

Our findings reveal a distinct enhancement in H_3_^+^ formation on nanoparticles with sharpened surface features, such as edges and facets, when exposed to strong local electric fields compared to smoother spherical geometries. While nanoparticle-mediated chemical reactivity has often been attributed to steric constraints and surface curvature, particularly in catalysis, our findings point toward a more dominant role played by surface charge localization. Specifically, the H_3_^+^ yield exhibits a nonlinear dependence on local charge density, indicating a field-mediated formation mechanism uniquely enabled by morphological inhomogeneity. The key distinction lies in the spatial confinement of charge at high-curvature regions, where enhanced local fields facilitate bond polarization, field-assisted tunneling, and directional proton migration, all contributing to significantly increased H_3_^+^ production.

H_3_^+^ formation has long been studied in gas-phase systems such as van der Waals clusters and interstellar environments, where it typically arises via ternary association of H_2_ and H^+^, stabilized by a third body.^14,15,35–37^ These thermodynamically governed reactions require multiple collisions and often yield a low efficiency under laboratory conditions. In larger molecular clusters, charge delocalization can further suppress H_3_^+^ formation by reducing Coulomb-driven bond rearrangements.^38^ Critically, gas-phase environments lack an interface that can stabilize or localize charge, offering limited opportunities for field-assisted bond manipulation.

More recently, dielectric nanoparticles have emerged as platforms to explore ion chemistry under strong-field conditions.^16^ On these surfaces, intense laser fields can induce localized charge regions that modestly enhance H_3_^+^ yields. However, the effects are typically constrained by the limited polarizability and field localization capacity in dielectric materials. In contrast, metallic gold nanoparticles exhibit strong field localization at high-curvature features, which substantially boost local fields even under off-resonant excitation and moderate fluences. Several prior works^17–19^ have shown that off-resonant strong-field excitation of plasmonic nanoparticles produces significant near-field enhancement and nonthermal ionization dynamics. While the fundamental dipolar plasmon resonance lies near 550–580 nm for ∼100 nm AuNPs, as shown in Figures S1c and S2c, the intense near-infrared fields used in this study drive strong-field ionization and charge localization that go beyond purely resonant plasmonic or thermal effects. Thermal desorption would lead to isotropic, broad distributions of the momentum distribution of the ionic fragments emitted from the nanoparticle surface, whereas field-driven charge localization produces anisotropic polarization-aligned momentum features, as observed in our measurements. The nonlinear scaling of H_3_^+^ yields with faceted geometries and electron counts observed in Figure 5c, as discussed in the upcoming section, provide direct evidence that the mechanism here is field-driven charge localization rather than thermal heating.

**Figure 5:**
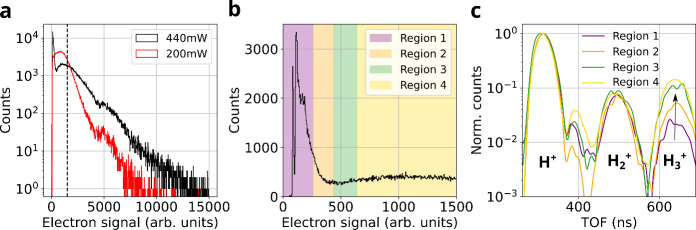
Hydrogen ion yields as a function of photoionization events. (a) Histogram of electron signal per laser shot at two excitation powers (200 mW, red; 440 mW, black), showing increased photoelectron emission and signal broadening with higher intensity. (b) Distribution of selected (subset indicated by black dashed line in (a)) detected electron signal intensities at 440 mW, binned into four regions representing increasing levels of laser field intensities. Region 1 corresponds to low-charge events, while Region 4 encompasses high-field, high-charge-density conditions. (c) Normalized time-of-flight (TOF) spectra of hydrogen fragment ions (H^+^, H_2_^+^, H_3_^+^) for each charge region. While H^+^ and H_2_^+^ show only moderate variation, a pronounced enhancement in the H_3_^+^ signal is observed in Regions 3 and 4 (green and yellow), indicating that H_3_^+^ formation is strongly favored under conditions of increased local electric fields.

To assess the role of surface charging more quantitatively, we analyzed ion yields as a function of coincident electron signal for 100 nm faceted AuNPs, as shown in Figure 5. The electron signal, taken as the integral of the channeltron output in our reaction nanoscope, provides a relative measure of the number of emitted electrons and thus serves as a semiquantitative proxy for local laser intensity.^39^ Although this signal cannot be directly mapped to absolute intensity due to the limited detector area, it reliably captures relative variations in the electron yield across the laser focal volume. As seen in Figure 5a, electron signal distributions measured at two different average powers (200 and 440 mW) exhibit a broad spread, reflecting the intensity inhomogeneity experienced by nanoparticles across the spatial Gaussian profile of the focus. The presence of a tail extending toward high electron counts, especially at 440 mW, indicates a subset of nanoparticles probing the peak intensities, consistent with expected local fields of 10^12^ to low 10^13^ W/cm^2^.^17,18^ However, the flattening of the histogram at high signals reflects the onset of saturation due to residual surface charge buildup, which suppresses further photoelectron emission, a well-known signature of strong-field photoemission from nanoparticles.^17,18^

We define four distinct regions of increasing electron signal, as illustrated in Figure 5b, corresponding to increasing local intensities and near-field strengths from Region 1 (low yield) to Region 4 (high yield). Each region thus represents a distinct interaction regime, with Region 1 dominated by low-field wings of the focus and Region 4 approaching the peak intensity region around the center of the Gaussian profile. The corresponding time-of-flight (TOF) spectra in Figure 5c reveal distinct ionization dynamics. While H^+^ and H_2_^+^ yields show only marginal variation across regions, the H_3_^+^ yield exhibits pronounced nonlinear growth, increasing by over an order of magnitude from Region 1 to Region 4. However, the convergence of H_3_^+^ yield between Regions 3 and 4, despite higher electron signals in the latter, indicates that H_3_^+^ formation also saturates at high local fields. This suggests that H_3_^+^ production, initially driven by increasing field strength and surface charge, becomes limited by residual charge dynamics, analogous to electron yield saturation. Mechanistically, H_3_^+^ enhancement at intermediate intensities likely reflects a transition to a field-assisted regime, where bond rearrangement and proton transfer are promoted by localized fields and growing surface charge. With increasing laser peak intensity, the accumulating positive residual charge creates a trapping field that inhibits the escape of subsequent low-energy electrons, thereby saturating the surface charge density.^17,18^ This, in turn, reduces the efficiency of the field-induced molecular rearrangement, ultimately capping the H_3_^+^ yield.

Such behavior is reminiscent of strong-field phenomena in condensed phase ionization, where increasing field gradients can distort potential energy surfaces and induce nonthermal reaction pathways.^1,40^ In the context of nanoparticles, we attribute the enhanced H_3_^+^ yield to the field-driven weakening of H–H bonds followed by directional proton migration, potentially mediated by adsorbate coupling and transient charge accumulation. Thus, our work expands the current understanding of strong-field-driven ion chemistry by demonstrating a surface-specific, charge-mediated reaction regime that cannot be explained by gas-phase collision dynamics or steric effects alone. It provides a new framework for investigating molecular reactivity at nanoparticle interfaces, especially under nonequilibrium conditions, where field gradients and charge localization emerge as active control parameters for driving chemical rearrangement.

In summary, this study establishes a direct link between surface charge localization and enhanced molecular reactivity on laser-irradiated AuNPs. By resolving H_3_^+^ formation across differently curved morphologies, we show that high-curvature regions on faceted particles not only concentrate charge more efficiently but also drive nonlinear proton rearrangement reactions, leading to preferential H_3_^+^ formation. These findings move beyond the established role of field enhancement at sharp features, highlighting charge density as a key parameter governing reaction efficiency. This charge-driven mechanism opens new pathways to control strong-field surface chemistry, with possible implications for astrochemical mimetics,^16,41^ field-assisted catalysis,^1^ and sustainable energy conversion pathways such as proton-coupled charge transfer in hydrogen fuel technologies.^42^

## Supplementary Material



## Notes

Characterization of faceted and spherical gold nanoparticles, including SEM images, dynamic light scattering spectra, UV–Vis absorption spectra, and tabulated physical and optical parameters (particle size, plasmon resonance, surface area, volume, concentration, and mass) (PDF)
