# Supplementary material for: Trihydrogen Formation on Gold Nanoparticles in Strong Laser Fields
Source: Nano Lett. 2026 Jan 27;26(5):1599–607. doi: 10.1021/acs.nanolett.5c03438 (PMC12904087; doi:10.1021/acs.nanolett.5c03438)
Supplement: Supplementary file 1 [file nl5c03438_si_001.pdf]

Supplementary Information: Tri-hydrogen formation on gold nanoparticles in strong laser fields

Ritika Dagar,[*,†,‡,¶] Wenbin Zhang,[†,§] Philipp Rosenberger,[†] Marcel Neuhaus,[†,¶] Boris Bergues,[†,‡] Cesar Costa Vera,[‖] and Matthias F. Kling[*,†,¶,⊥]

†*Department of Physics, Ludwig-Maximilians-Universität Munich, D-85748, Garching, Germany*

‡*Max Planck Institute of Quantum Optics, D-85748, Garching, Germany*

¶*Stanford PULSE Institute, SLAC National Accelerator Laboratory, Menlo Park, CA, 94025, USA*

§*State Key Laboratory of Precision Spectroscopy, East China Normal University, Shanghai, 200241, China*

‖*Department of Physics, Escuela Politécnica Nacional, Quito, 170525, Ecuador*

⊥*Applied Physics Department, Stanford University, Stanford, CA, 94305, USA*

E-mail: rdagar@stanford.edu; kling@stanford.edu

Nanoparticle Characterization

Faceted Gold Nanoparticles

The faceted nanoparticles were purchased commercially from Sigma-Aldrich (product no. 742031). The AuNPs were characterized by dynamic light scattering (DLS) and UV–Vis absorption spectroscopy. DLS measurements yielded a hydrodynamic size of 113 nm with a polydispersity index (PDI) of 0.04, indicating a narrow size distribution (Figure S1a). The optical response was probed by UV–Vis spectroscopy, which showed a peak surface plasmon resonance (SPR) at 572 nm (Figure S1b). Derived physical parameters are summarized in Table S1, including particle volume (5.24×10^5 nm^3), surface area (3.14×10^4 nm^2), and surface-to-volume ratio (0.06). The nanoparticle concentration was 3.84×10^9 particles/mL, with a mass concentration of 0.039 mg/mL, as determined from the supplier's specifications.

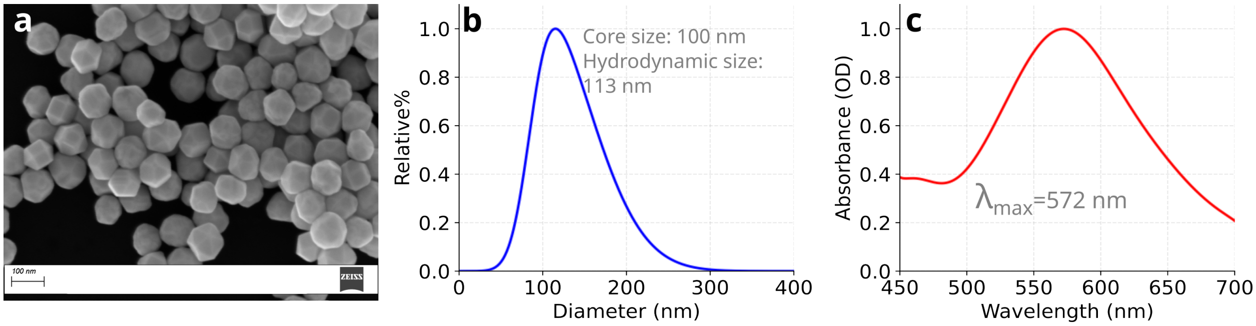

Figure S1: **Faceted AuNPs (a)** SEM images of the 100 nm faceted AuNPs**(b)** Dynamic light scattering (DLS) of 100 nm faceted AuNPs showing a narrow distribution with PDI = 0.04. **(c)** UV–Vis spectrum with a plasmon resonance maximum at 572 nm.

Table 1: Physical and optical parameters of faceted AuNPs (100 nm) as provided by Sigma-Aldrich

Parameter	Value
Diameter (nm)	100
Peak SPR (nm)	572
Particle Concentration (NPs/mL)	3.84×10^9
Mass Concentration (mg/mL)	0.039
Molar Extinction ($M^{-1}cm^{-1}$)	1.57×10^{11}
Surface Area (nm^2)	3.14×10^4
Volume (nm^3)	5.24×10^5
Surface/Volume Ratio	0.06
Particle Mass (g)	1.02×10^{-14}
Molar Mass (g/mol)	6.11×10^4
Molar Concentration (M)	6.37×10^{-12}

Gold Nanospheres

For comparison, spherical gold nanoparticles (100 nm, citrate-stabilized) were purchased from nanoComposix (AUCN100). Transmission electron microscopy (TEM) determined a mean diameter of 103 ± 10 nm, with a hydrodynamic diameter of 105 nm and coefficient of variation of 9.4%. The zeta potential was –54 mV, confirming colloidal stability. UV–Vis absorption spectroscopy revealed a plasmon resonance maximum at 563 nm (Figure S2a). The particle concentration was 4.7×10^9 particles/mL with a gold purity of 99.99% and a mass concentration of 0.052 mg/mL, as specified by the manufacturer.

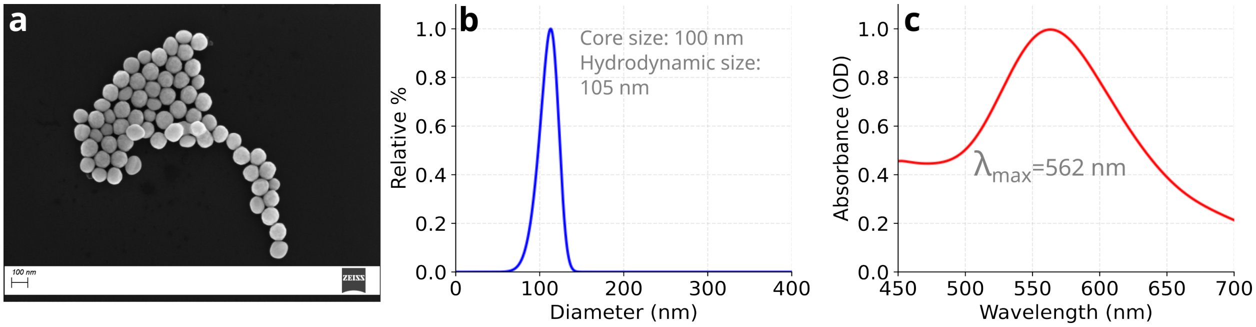

Figure S2: **Faceted AuNPs (a)** SEM images of the 100 nm faceted AuNPs**(b)** Dynamic light scattering (DLS) of 100 nm faceted AuNPs showing a narrow distribution with PDI = 0.04. **(c)** UV–Vis spectrum with a plasmon resonance maximum at 572 nm.

Table 2: Physical and optical parameters of 100 nm citrate-stabilized Au nanospheres as provided by nanoComposix

Parameter	Value
Diameter (TEM, nm)	103 ± 10
Hydrodynamic Diameter (nm)	105
Coefficient of Variation (%)	9.4
Zeta Potential (mV)	–54
Plasmon Resonance λ_{max} (nm)	563
Particle Concentration (mL^{-1})	4.7×10^9
Mass Concentration (mg/mL)	0.052
Surface Area (m^2/g)	3.0
pH	7.6
